# Restoration of Hair Luster via Novel Biomarker COL7A1 by Minoxidil, Caffeine, and Biotin

**DOI:** 10.3390/cimb47060468

**Published:** 2025-06-18

**Authors:** Ngoc Ha Nguyen, Young In Lee, Hyeon-Ah Do, Inhee Jung, Jae Hyun Park, Sung Jun Lee, Ju Hee Lee

**Affiliations:** 1 Department of Dermatology & Cutaneous Biology Research Institute, Yonsei University College of Medicine, Seoul 03722, Republic of Korea; nguyenngocha7996@gmail.com (N.H.N.); ylee1124@yuhs.ac (Y.I.L.); 2 Department of Dermatology, University of Medicine and Pharmacy at Ho Chi Minh City, Ho Chi Minh City 17000, Vietnam; 3 Scar Laser and Plastic Surgery Center, Yonsei Cancer Hospital, Seoul 03722, Republic of Korea; 4 Global Medical Research Center Co., Ltd., Seoul 06526, Republic of Korea; dha0201@gmrc.co.kr (H.-A.D.); ihjung@gmrc.co.kr (I.J.); 5 DANA Plastic Surgery, Seoul 06038, Republic of Korea; jay8384@naver.com; 6 Liting Plastic Surgery, Seoul 06035, Republic of Korea; justdisj@naver.com

**Keywords:** *COL7A1*, hair luster, *KRTAP*, minoxidil, biotin, caffeine

## Abstract

Hair luster, a key component of visual hair quality, depends largely on the integrity of the cuticle. While cosmetic products offer temporarily enhanced luster, their effects are limited due to a poor understanding of the underlying molecular mechanisms. In this study, we employed a UVB-induced mouse model of hair luster loss to identify differentially expressed genes via quantitative real-time reverse transcription PCR. Key candidate genes were subsequently validated in vitro using human hair follicle dermal papilla cells and in ex vivo human scalp hair follicle tissue models. Subsequently, we evaluated the effects of minoxidil, caffeine, and biotin on gene expression and luster restoration. UVB exposure suppressed several luster-related genes, with *COL7A1* consistently downregulated across all models. Treatment with minoxidil, caffeine, and biotin restored the expression of *COL7A1* along with *KRTAP5-5*, *KRTAP5-4*, *TGM3*, and *PTK7*. These findings highlight *COL7A1* as a novel molecular marker for hair luster and support its modulation as a potential therapeutic strategy.

## 1. Introduction

Hair plays a vital role in both personal appearance and dermatological assessment, reflecting an individual’s overall health and grooming. Clinicians often evaluate characteristics such as shaft diameter, elasticity, and scalp conditions—like pH and erythema—to assess hair integrity [1,2]. Among these features, hair luster, defined as the ability of hair fibers to reflect light, stands out as a key determinant of visual appeal [3]. It is primarily influenced by the structural quality and smoothness of the cuticle, the hair’s outermost layer [4]. The cuticle comprises overlapping flattened cells, each of which possesses multiple layers, namely the epicuticle, A-layer, exocuticle, endocuticle, and cell membrane complex (CMC) [5]. The rich cysteine component in the A-layer and exocuticle contributes to the mechanical resilience of the hair fiber via abundant disulfide bonds. The CMC, meanwhile, links individual cuticle cells together [5]. The outermost membrane, known as the epicuticle, is structurally constituted by keratin-associated proteins (KRTAP), a family of high-sulfur proteins cross-linked together, promoting cuticle cohesion and smoothness [6,7,8]. In addition, 18-methyleicosanoic acid (18-MEA), the predominant lipid component of the epicuticle covalently bound to the cuticle by thioester linkages, covers the entirety of this layer [9]. This fatty acid offers the first protective hydrophobic barrier for hair luster against external insults and reduces friction between hair fibers to prevent physical wear [9]. However, hair luster is still highly susceptible to damage from environmental and lifestyle factors, including ultraviolet (UV) radiation, chemical exposure, mechanical trauma, and nutritional deficiencies [10]. Ultraviolet B (UVB) radiation, in particular, causes major morphological alterations to the cuticle compared to UVA, due to its concentrated impact on more superficial levels [11,12]. UVB disrupts the protein architecture of the cuticle via rupture of disulfide bonds, leading to surface irregularities and porosity that reduce light reflectivity and luster [9,12,13]. UVB-induced loss of the 18-MEA layer is also attributable to this consequence [14].

To counteract the detrimental effects on hair luster, numerous cosmetic treatments, mainly shampoos and conditioners, have been developed and commercialized on the market as solutions. However, their effects are often temporary, resulting from the filling of surface fractures or chemically flattening the cuticle, and do not address the underlying biological mechanisms [9,15]. Thus, there is an unmet need to develop lasting treatments that restore luster through molecular-level repair and regulation. Yet, the molecular basis of hair luster remains poorly understood, with few validated genetic targets. This limits the development of durable, targeted therapies.

To address this gap, we designed a comprehensive study employing in vitro, ex vivo, and in vivo models to identify and validate novel molecular factors involved in hair luster regulation. Using our previously described UVB-induced hair luster loss mouse model [6], we identified candidate genes that were further validated in human hair follicle dermal papilla cells (HFDPCs) and human scalp hair follicle tissues. Additionally, this study investigated whether minoxidil, biotin, and caffeine—compounds commonly used in dermatology—could restore luster at the gene expression level. We hypothesize that these treatments may reverse UVB-induced changes and upregulate key genes contributing to cuticle integrity and light reflectivity.

This study aims to broaden our understanding of the genetic regulation of hair luster and provide a molecular framework for future interventions targeting both cosmetic outcomes and follicular health.

## 2. Materials and Methods

### 2.1. In Vivo UVB-Induced Hair Luster Loss Mouse Model

Our previously established mouse model demonstrated a significant reduction in hair luster after UVB irradiation and its restoration after oral treatment with minoxidil [6]. Regarding the assessment of hair luster, we utilized the Investigator’s Global Assessment (IGA) scale and computer-based quantification of luminous pixels on photographs [6]. Specifically, the IGA scale consisted of 5 categories: −2, extremely rough; −1, rough; 0, normal; 1, lustrous; and 2, extremely lustrous. For photographs, we used a dedicated light source with an intensity of 650 lux to illuminate the skin sample mounted on a rod. We took photos with a camera strategically placed 40 cm away from the sample, forming a 70-degree angle with the light source for optimal lighting. Additionally, photographic parameters were rigorously controlled: the camera’s shutter speed was 1/125, with an aperture of f/5.6, International Organization for Standardization sensitivity of 200, and an F-number of 10. We then used the I-MAXPLUS software (v1.0) to measure hair luster by detecting shiny regions in photographs, quantifying both their area and brightness, and expressing the results in pixel values. Based on these methods and findings, our study utilized a similar protocol to induce hair luster loss. Five-week-old male C57BL/6 mice (Orient Bio, Seongnam, Republic of Korea) were used to establish a UVB-induced model of hair luster loss. We randomly assigned 15 mice to 3 groups (n = 5 per group): control, UVB-irradiated, and UVB-irradiated with minoxidil treatment. We grouped 3 to 5 mice per cage under a controlled 12 h light/dark cycle, with unrestricted access to standard chow and water. All procedures followed the guidelines established by the Association for Assessment and Accreditation of Laboratory Animal Care International. Prior to the start of the experiment, the animals were given a 7-day acclimation period to adjust to the housing conditions. When necessary (during hair shaving or UVB exposure), we sedated mice using inhalational anesthesia with isoflurane (1.5–2%) (N01AB06, Hana Pharm, Seoul, Republic of Korea).

**Figure 1:**
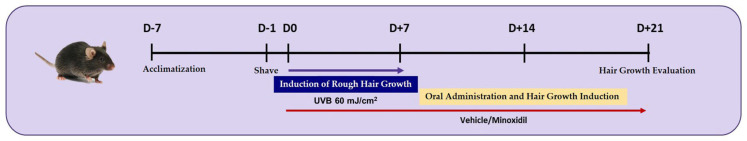
The procedure and timeline of the UVB-induced hair luster loss in an in vivo mouse model and minoxidil treatment. D, day; UVB, ultraviolet B.

One day before starting the experiments, the dorsal hair of mice was shaved with hair clippers to limit trauma. At the start of the experiment, UVB irradiation was performed to induce hair luster loss, using a 312 nm UV lamp (BLX312 UV Cross-linker, Vilber Lourmat, Marne-la-Vallée, Paris, France) at a dose of 60 mJ/cm^2^ per day for 7 consecutive days, yielding a cumulative dose of 420 mJ/cm^2^. In the treatment group, minoxidil (Sigma-Aldrich, St. Louis, MO, USA) was administered orally via gavage at a dose of 0.5 mg/kg once daily for 3 weeks. Fourteen days after the final UVB exposure, we collected 5 mm punch biopsies of skin samples. Next, we homogenized the tissues using a TissueLyser (Qiagen, Hilden, Germany), then extracted RNA and synthesized complementary DNA (cDNA). Finally, we performed quantitative real-time reverse transcription polymerase chain reaction (qRT-PCR) to quantify the expression levels of 10 hair luster–associated genes (Table 1). The procedure is summarized in Figure 1.

### 2.2. In Vitro and Ex Vivo Experiments

#### 2.2.1. In Vitro Human Hair Follicle Dermal Papilla Cell Culture

We cultured HFDPCs (ATCC, Manassas, VA, USA) in Human Follicle Dermal Papilla Cell Growth Medium (Promocell, Heidelberg, Germany), containing fetal calf serum, bovine pituitary extract, basic fibroblast growth factor, insulin, and 1% penicillin-streptomycin (Gibco, Waltham, MA, USA). We maintained cultures at 37 °C in a humidified 5% CO_2_ atmosphere. We then used the cells in passage 5.

#### 2.2.2. Ex Vivo Human Hair Follicles Culture

We acquired post-surgical human scalp tissues from 5 donors and extracted 10 hair follicles from each donor’s tissue. Specifically, we washed the tissues with phosphate-buffered saline and dissected them under a stereomicroscope (Stemi 508, Zeiss, Oberkochen, Germany) to isolate individual hair follicles. Then, we cultured the follicles in William’s Medium E (Sigma-Aldrich) supplemented with 2 mM L-glutamine (Sigma-Aldrich), 10 μg/mL insulin (Sigma-Aldrich), 100 ng/mL hydrocortisone (Sigma-Aldrich), 0.1% fungizone (Gibco), 1% antibiotic-antimycotic (Gibco), and 1% penicillin-streptavidin (Gibco). Finally, we incubated the cultures at 37 °C in a 5% CO_2_ environment.

#### 2.2.3. UVB Irradiation and Treatment

For the in vitro study, we seeded HFDPCs at a density of 5 × 10^4^ cells per well in 6-well plates and cultured until 80% confluence. We irradiated the cells with 60 mJ/cm^2^ UVB using the UV-crosslinker (Vilber Lourmat) to induce luster loss and subsequently treated them with minoxidil (5 μM), caffeine (40 ppm), or biotin (30 μg/mL) in a serum-free medium. We then collected the cells and supernatants after 24 h. For the ex vivo study, we irradiated isolated hair follicles with 60 mJ/cm^2^ UVB and treated them with the same compounds in the same doses. We refreshed the culture medium every 2 days and harvested the follicles after 12 days.

### 2.3. RNA Extraction and qRT-PCR

We extracted total RNA from HFDPCs and human hair follicles using TRIzol reagent (Invitrogen, Waltham, MA, USA), and reverse-transcribed into cDNA using the RNA to cDNA EcoDry™ Premix (Clontech, Mountain View, CA, USA). qRT-PCR was performed using the TaqMan Fast Advanced Master Mix (Applied Biosystems, Carlsbad, CA, USA). The primers used in the experiments are listed in Table 1. Relative quantification of gene expression levels was calculated using the 2−ΔΔC_T_ method, based on the cycle threshold (C_T_) values obtained from qRT-PCR. We independently performed each cell experiment at least three times.

### 2.4. Ethics

The procedures used and the care of animals were approved by the Institutional Animal Care and Use Committee at Yonsei University (IACUC No. 2022-0267).

Human scalp tissue experiments were approved by the Global Medical Research Center Institutional Review Board (approval No. GIRB-24912-FW).

### 2.5. Statistical Analysis

All results obtained from the experiment were analyzed as the mean and standard error of at least 3 independent experiments and were verified using the IBM SPSS Statistics 25.0 program. The significance of the experimental group and the control group was determined through an independent samples t-test without multiple-comparison correction. The significance level was set at *p* < 0.05.

## 3. Results

### 3.1. Gene Expression Analysis in Skin Tissue of the UVB-Induced Hair Luster Mouse Model

**Figure 2:**
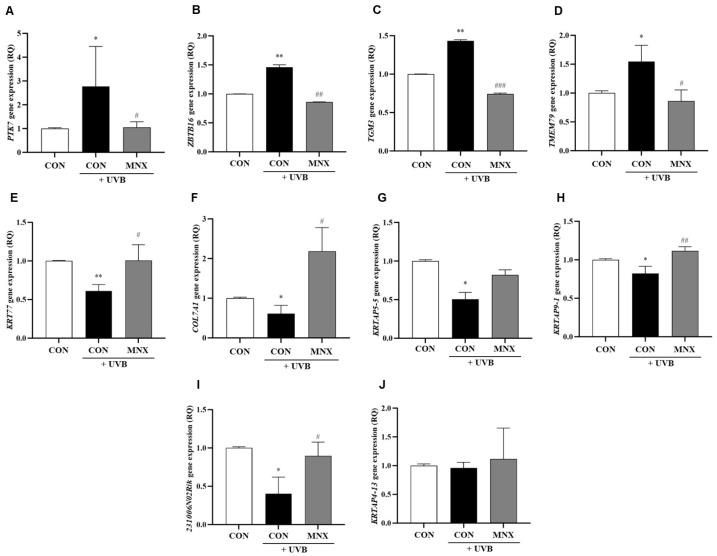
Gene expression analysis results via qRT-PCR in the hair luster mouse model. (**A–J**) Relative mRNA expression levels of hair luster-related genes *PTK7*, *ZTBT16*, *TGM3*, *TMEM79*, *KRT77*, *COL7A1*, *KRTAP5-5*, *KRTAP9-1*, *231006N02Rik*, and *KRTAP4-13* were measured in a hair luster mouse model. All experimental groups were conducted with n = 5. * *p* < 0.05, ** *p* <0.01 vs. CON. # *p* < 0.05, ## *p* < 0.01, ### *p* < 0.001 vs. UVB. CON, control group; UVB, ultraviolet B 60 mJ/cm^2^ irradiated group; MNX, minoxidil 5 mg/kg/day treated group.

A previous next-generation sequencing (NGS) analysis identified 10 genes associated with hair luster that were differentially expressed following UVB irradiation (Table S1) [6]: *PTK7*, *ZBTB16*, *KRTAP4-13*, *KRTAP5-5*, *TGM3*, *TMEM79*, *KRT77*, *COL7A1*, *KRTAP9-1*, and *2310061N02Rik*. To validate these findings, we performed qRT-PCR on skin tissue samples from the mouse model. The expression levels of *PTK7*, *ZBTB16*, *TGM3*, and *TMEM79* were significantly elevated in the UVB-irradiated group compared to controls, with subsequent minoxidil treatment (UVB-MNX group) resulting in marked reductions (*p* < 0.05, Figure 2A–D). Conversely, *KRT77*, *COL7A1*, *KRTAP5-5*, *KRTAP9-1*, and *2310061N02Rik* expression levels were significantly decreased in the UVB-irradiated group relative to controls, but restored in the UVB-MNX group (*p* < 0.05, Figure 2E–I). *KRTAP4-13* expression remained unchanged (*p* > 0.05, Figure 2J). Based on these results, six genes (*PTK7*, *TGM3*, *COL7A1*, *KRTAP5-5*, *KRTAP5-4*, and *KRTAP4-4*) were selected for further investigation in human in vitro and ex vivo models.

### 3.2. Gene Expression Analysis in Human Hair Follicle Dermal Papilla Cells

**Figure 3:**
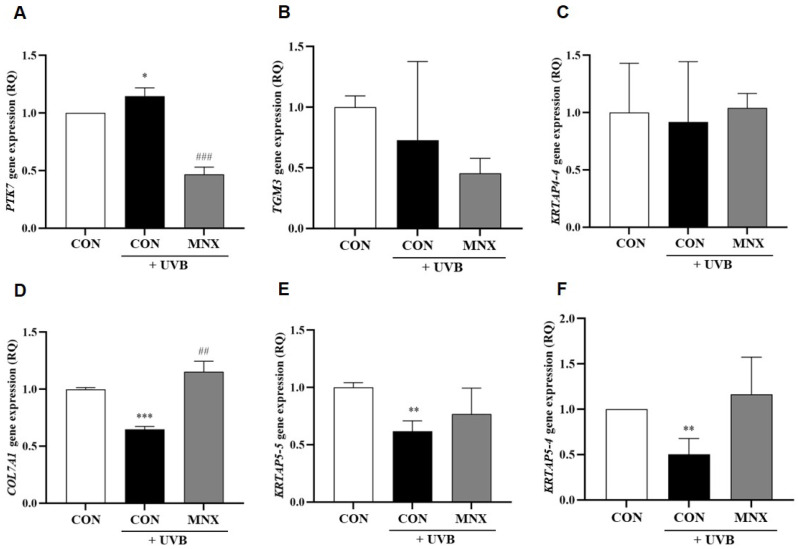
Gene expression analysis results via qRT-PCR in hair follicle dermal papilla cells. (**A**–**F**) Relative mRNA expression levels of selected hair luster-related genes, including *PTK7*, *TGM3*, *KRTAP4-4*, *COL7A1*, *KRTAP5-5*, and *KRTAP5-4*, were measured in HFDPCs. All experimental groups were conducted with n = 3. * *p* < 0.05, ** *p* < 0.01, *** *p* < 0.001 vs. CON. ## *p* < 0.01, ### *p* < 0.001 vs. UVB. CON, control group; UVB, ultraviolet B 60 mJ/cm^2^ irradiated group; MNX, minoxidil 5 μM treated group.

**Figure 4:**
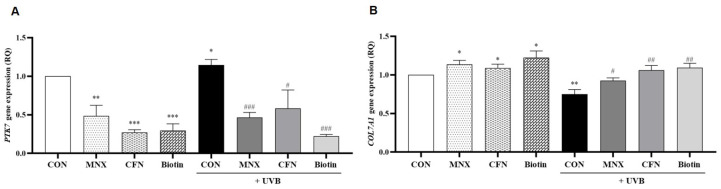
Confirmation of *PTK7* and *COL7A1* gene expression level via qRT-PCR in hair follicle dermal papilla cells (**A**,**B**). All experimental groups were conducted with n = 3. * *p* < 0.05, ** *p* < 0.01, *** *p* < 0.001 vs. CON. # *p* < 0.05, ## *p* < 0.01, ### *p* < 0.001 vs. UVB. CON, control group; UVB, ultraviolet B 60 mJ/cm^2^ irradiated group; MNX, minoxidil 5 μM treated group; CFN, caffeine 40 ppm treated group; Biotin, biotin 30 μg/mL treated group.

In HFDPCs, UVB irradiation significantly upregulated *PTK7* expression, which was subsequently reversed with minoxidil treatment (*p* < 0.05, Figure 3A). The expression of *TGM3* and *KRTAP4-4* was unaffected by UVB exposure (*p* > 0.05, Figure 3B,C). In contrast, *COL7A1*, *KRTAP5-5*, and *KRTAP5-4* were significantly downregulated in the UVB-irradiated group and restored with minoxidil treatment (*p* < 0.05, Figure 3D–F). Additional experiments evaluated the effects of caffeine (UVB-CFN group) and biotin (UVB-Biotin group). *PTK7* expression, elevated by UVB, was significantly reduced with both treatments (*p* < 0.05, Figure 4A). Conversely, *COL7A1* expression, suppressed by UVB, was significantly increased following caffeine and biotin treatment (*p* < 0.05, Figure 4B).

### 3.3. Gene Expression Analysis in Human Hair Follicle Tissue

**Figure 5:**
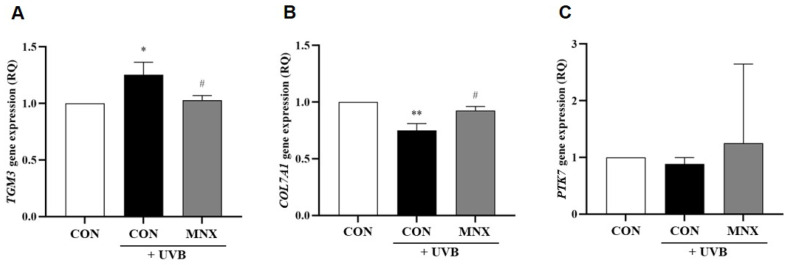
Gene expression analysis results via qRT-PCR in human hair follicle tissue. (**A**–**C**) Relative RNA expression levels of selected hair luster-related genes, including *TGM3*, *COL7A1*, and *PTK7*, were measured in human hair follicle tissues. All experimental groups were conducted with n = 3. * *p* < 0.05, ** *p* < 0.01 vs. CON. # *p* < 0.05 vs. UVB. CON, control group; UVB, ultraviolet B 60 mJ/cm^2^ irradiated group; MNX, minoxidil 5 μM treated group.

**Figure 6:**
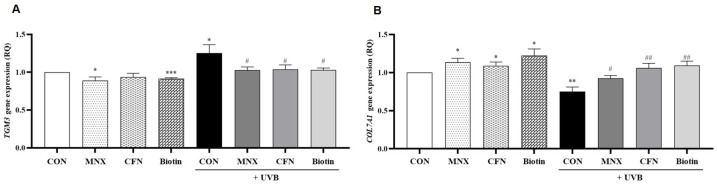
Confirmation of *TGM3* and *COL7A1* gene expression levels via qRT-PCR in human hair follicle tissues (**A**,**B**). All experimental groups were conducted with n = 3. * *p* < 0.05, ** *p* < 0.01, *** *p* < 0.001 vs. CON. # *p* < 0.05, ## *p* < 0.01 vs. UVB. CON, control group; UVB, ultraviolet B 60 mJ/cm^2^ irradiated group; MNX, minoxidil 5μM treated group; CFN, caffeine 40 ppm treated group; Biotin, biotin 30 μg/mL treated group.

In the human hair follicle tissue model, UVB irradiation resulted in a significant increase in *TGM3* expression, which was reversed by minoxidil (*p* < 0.05, Figure 5A). *COL7A1* expression was markedly reduced by UVB irradiation and restored in the UVB-MNX group (*p* < 0.05, Figure 5B). *PTK7* did not show significant changes in this model (*p* > 0.05, Figure 5C). Further analysis revealed that both caffeine and biotin treatments significantly decreased *TGM3* expression and increased *COL7A1* expression compared to the UVB-irradiated group (*p* < 0.05, Figure 6A,B).

## 4. Discussion

To investigate molecular regulators of hair luster, this study used our previously described UVB-induced luster loss model [6] and validated gene expression changes in mice, human hair follicle dermal papilla cells, and human scalp hair follicle tissue. This multi-model approach enabled cross-species comparison of UVB effects and treatment responses. Mechanistically, UVB radiation is known to degrade the 18-MEA layer of the epicuticle and create microscopic cuticle pits on the hair fiber surface, which leads to increased light scattering and a reduction in hair luster [9,12,13,14]. As shown in our study, UVB exposure disrupted the expression of most target genes. Similar downregulation patterns were observed in human models, particularly for *COL7A1*. In subsequent experiments, treatment with minoxidil, caffeine, or biotin restored expression levels of *COL7A1*, *PTK7*, *TGM3*, and *KRTAP* family genes. These treatment effects suggest a conserved mechanism of gene modulation across models.

The consistent downregulation of *COL7A1* after UVB exposure underscores its importance in maintaining hair luster. This gene encodes type VII collagen, a key extracellular matrix protein that anchors the epidermal–dermal junction through interactions with laminins and integrins [16,17]. Within hair follicles, these anchoring fibrils form part of the basement membrane zone (BMZ) between the dermal papilla and hair matrix. This zone supports cell communication and nutrient exchange between dermal fibroblasts and matrix epithelial cells [18,19,20]. These cells later differentiate into the cuticle and cortex, which determine hair strength and luster [18,19,20]. Additionally, clinical conditions, namely dystrophic epidermolysis bullosa and epidermolysis bullosa acquisita, which involve *COL7A1* mutations or autoantibodies, often manifest as alopecia and brittle hair [21,22,23]. Therefore, replenishing type VII collagen may strengthen the connection between the cuticle and the follicle base, helping to stabilize the hair shaft and enhance its smoothness as well as light reflectivity. Additionally, one other possible mechanism by which collagen may restore hair luster is by enhancing the disulfide bonds to reduce hair structure disruption [24]. Several studies have shown that oral collagen supplementation improves hair luster in aged mice and human subjects [25,26]. As a whole, these associations further highlight *COL7A*′s role in hair fiber integrity through BMZ regulation and restoration of protein linkage. Nevertheless, additional mechanistic studies are needed to fully explore *COL7A1*’s function and therapeutic potential in hair luster restoration.

The altered expression of *KRTAP* genes in HFDPCs suggests that these proteins also contribute to hair luster regulation. The hair shaft consists of an outer cuticle, a keratin-rich cortex, and a central medulla [27,28]. Members of the *KRTAP5* family are primarily expressed in the epicuticle and promote structural integrity and surface smoothness by forming disulfide bonds with keratin filaments [7,8]. In contrast, *KRTAP4*, *KRTAP9*, and *KRTAP13* are localized to the cortex [27]. The UVB-induced downregulation of *KRTAP5* genes without significant changes in cortical *KRTAPs* is consistent with their localization and specific roles in maintaining cuticle structure and luster. Furthermore, its post-treatment restoration suggests that *KRTAP5* is a possible molecular treatment target for future hair care formulations.

*TGM3*, an enzyme that cross-links keratin filaments and proteins like trichohyalin, showed increased expression in response to UVB-induced damage in our ex vivo model [29]. Interestingly, loss-of-function mutations in *TGM3* are linked to uncombable hair syndrome, a condition characterized by unruly yet lustrous hair [29]. This contrast suggests that excessive *TGM3* expression may reduce luster, possibly by over-stabilizing structural proteins. Additionally, *PTK7*, a regulator of the Wnt planar cell polarity pathway, also showed altered expression after UVB irradiation. Although its role in scalp hair remains unclear, the observed changes indicate a potential function in follicular regulation [30].

Our findings further clarify the molecular actions of minoxidil, caffeine, and biotin—agents traditionally used to promote hair growth. These compounds reversed UVB-induced influence of key structural genes, including *COL7A1*, *KRTAP5-4*, *KRTAP5-5*, *TGM3*, and *PTK7*. While their known mechanisms involve anagen phase induction and metabolic stimulation, our data indicate they may also enhance hair luster by restoring genes associated with extracellular matrix integrity and cuticle cohesion [31,32,33]. These insights expand the therapeutic relevance of these compounds beyond hair regeneration and into the domain of aesthetic enhancement, suggesting translational opportunities for developing biologically based cosmeceuticals that target hair quality at the molecular level.

This study’s strength lies in its comprehensive use of in vitro, ex vivo, and in vivo models, allowing for translational relevance. Moreover, the evaluation of widely used therapeutic agents offers clinically applicable insights. However, while this study provides compelling molecular evidence, it is subject to several limitations. Firstly, our conclusions are based on mRNA expression levels. Corresponding changes in protein levels were not confirmed. Future studies should incorporate proteomic analyses and immunohistochemical validation to strengthen mechanistic insights. Secondly, although we used human-derived dermal papilla cells and ex vivo follicles, these models do not fully recapitulate the complexity of the human scalp environment. Clinical studies will be necessary to assess the actual impact of these treatments on hair luster in diverse populations. Additionally, although some findings showed statistical significance, we did not apply false discovery rate correction due to the study’s exploratory nature and limited sample size. Conducting such corrections in this context could be overly conservative, potentially obscuring meaningful changes in gene expression. To validate these preliminary observations, future studies involving larger cohorts or independent datasets are essential. Lastly, the scope of compounds tested was limited to only three agents. Broader screening of additional compounds—including peptides, retinoids, and botanical extracts—may uncover new candidates for targeted luster enhancement.

Despite limitations, by identifying *COL7A1* as a molecular marker associated with cuticle integrity, this study facilitates the development of targeted treatments aimed at improving hair luster through biological repair rather than surface coating. This approach may enable longer-lasting results than traditional cosmetic products, offering therapeutic benefits for individuals with hair dullness due to environmental stress or aging. Moreover, *COL7A1* and its related pathways could serve as biomarkers in future clinical trials evaluating the efficacy of novel luster-enhancing formulations.

## 5. Conclusions

In conclusion, this study provides valuable insights into the genetic mechanisms regulating hair luster by integrating in vitro, ex vivo, and in vivo models. By identifying key genes such as *KRTAP5*, *TGM3*, *PTK7*, and particularly *COL7A1*, which are significantly affected by UVB-induced damage and restored by minoxidil, caffeine, and biotin, this research highlights potential molecular targets for enhancing hair luster. Further studies involving clinical trials and broader genetic analyses will be essential to validate these findings and explore their practical applications in dermatology and hair care.

## Figures and Tables

**Table 1:** List of primers.

Species	Primer Name	Cat. No
Human	*PTK7*	Hs00897151_m1
	*TGM3*	Hs00897151_m1
	*KRTAP4-4*	Hs00540375_s1
	*COL7A1*	Hs00164310_m1
	*KRTAP5-5*	Hs03037438_s1
	*KRTAP5-4*	Hs04195613_s1
	*GAPDH*	Hs02786624_g1
Mouse	*PTK7*	Mm00613362_m1
	*ZTBT16*	Mm01176868_m1
	*TGM3*	Mm01268669_m1
	*TMEM79*	Mm00470361_m1
	*KRTAP4-13*	Mm00471879_s1
	*KRT77*	Mm02343482_m1
	*COL7A1*	Mm01227938_m1
	*KRTAP5-5*	Mm03015615_s1
	*KRTAP9-1*	Mm00497172_s1
	*231006N02Rik*	Mm03991538_s1
	*GAPDH*	Mm99999915_g1

## Data Availability

The original contributions presented in this study are included in the article/Supplementary Materials. Further inquiries can be directed to the corresponding author.

## Supplementary Materials

The following supporting information can be downloaded at https://www.mdpi.com/article/10.3390/cimb47060468/s1, Table S1. Hair luster-related genes were selected through NGS analysis in the previous study. References [34,35,36,37,38,39,40,41,42] are cited in the Supplementary Materials.

## Abbreviations

The following abbreviations are used in this manuscript:

BMZ	Basement Membrane Zone
COL7A1	Collagen Type VII Alpha 1
cDNA	Complementary DNA
DNA	Deoxyribonucleic Acid
HFDPCs	Human Hair Follicle Dermal Papilla Cells
IACUC	Institutional Animal Care and Use Committee
IRB	Institutional Review Board
KRTAP	Keratin-Associated Protein
NGS	Next-Generation Sequencing
PTK7	Protein Tyrosine Kinase 7
qRT-PCR	Quantitative Real-Time Reverse Transcriptase Polymerase Chain Reaction
RNA	Ribonucleic Acid
TGM3	Transglutaminase 3
TMEM79	Transmembrane Protein 79
UV	Ultraviolet
UVB	Ultraviolet B
