# Supplementary material for: Restoration of Hair Luster via Novel Biomarker COL7A1 by Minoxidil, Caffeine, and Biotin
Source: Curr Issues Mol Biol. 2025 Jun 18;47(6):468. doi: 10.3390/cimb47060468 (PMC12191631; doi:10.3390/cimb47060468)
Supplement: Supplementary file 1 [file cimb-47-00468-s001.zip › cimb-3668970-supplementary.pdf]

Table S1. Hair luster-related genes selected through NGS analysis in the previous study [1].

Gene	NGS Analysis (Compared to Negative Control)	Fold Change	*p*-Value	Function
PTK7	Increased	4.661	0.000	Gene related to Wnt/β-catenin signaling and ERK pathway can affect cell proliferation [2]
ZBTB16	Decreased	0.180	0.001	Gene can regulate cell cycle and inhibit cell proliferation [3]
KRTAP4-13	Increased	5.051	0.017	Associated with keratin [4]
KRTAP5-5	Increased	2.856	0.011	Essential for the formation of a rigid and resistant hair shaft through their extensive disulfide bond cross-linking with abundant cysteine residues of hair keratins [4]
TGM3	Increased	2.599	0.022	Associated with Uncombable hair syndrome [5]
TMEM79	Increased	1.299	0.014	If there is a defect in the lamellar granule component, it can cause matted hair and dryness such as atopic dermatitis [6]
KRT77	Decreased	0.408	0.021	Associated with keratin [7]
COL7A1	Decreased	0.380	0.036	It is associated with collagen production and causes skin dryness when insufficient [8,9]
KRTAP9-1	Increased	2.822	0.022	Associated with keratin [4]
KRTAP13-22	Increased	3.690	0.040	Keratinization [10]

References

1. Chung, K.B.; Lee, Y.I.; Kim, Y.J.; Do, H.A.; Suk, J.; Jung, I.; Kim, D.Y.; Lee, J.H. Quantitative Analysis of Hair Luster in a Novel Ultraviolet-Irradiated Mouse Model. *Mol. Sci.* **2024**, *25*, 1885. https://doi.org/10.3390/ijms25031885.
2. Ji, J.; Qian, Q.; Cheng, W.; Ye, X.; Jing, A.; Ma, S.; Ding, Y.; Ma, X.; Wang, Y.; Sun, Q.; et al. FOXP4-mediated induction of PTK7 activates the Wnt/beta-catenin pathway and promotes ovarian cancer development. *Cell Death Dis.* **2024**, *15*, 332.
3. Wang, K.; Guo, D.; Yan, T.; Sun, S.; Wang, Y.; Zheng, H.; Wang, G.; Du, J. ZBTB16 inhibits DNA replication and induces cell cycle arrest by targeting WDHD1 transcription in lung adenocarcinoma. *Oncogene* **2024**, *43*, 1796–1810.
4. Wu, D.D.; Irwin, D.M.; Zhang, Y.P. Molecular evolution of the keratin associated protein gene family in mammals, role in the evolution of mammalian hair. *BMC Evol. Biol.* **2008**, *8*, 241.
5. Chermnykh, E.S.; Alpeeva, E.V.; Vorotelyak, E.A. Transglutaminase 3: The Involvement in Epithelial Differentiation and Cancer. *Cells* **2020**, *9*, 1996.
6. Sasaki, T.; Shiohama, A.; Kubo, A.; Kawasaki, H.; Ishida-Yamamoto, A.; Yamada, T.; Hachiya, T.; Shimizu, A.; Okano, H.; Kudoh, J.; et al. A homozygous nonsense mutation in the gene for Tmem79, a component for the lamellar granule secretory system, produces spontaneous eczema in an experimental model of atopic dermatitis. *J. Allergy Clin. Immunol.* **2013**, *132*, 1111–1120.e4.
7. Hinbest, A.J.; Eldirany, S.A.; Ho, M.; Bunick, C.G. Molecular Modeling of Pathogenic Mutations in the Keratin 1B Domain. *Int. J. Mol. Sci.* **2020**, *21*, 6641.
8. Rouanet, S.; Warrick, E.; Gache, Y.; Scarzello, S.; Avril, M.F.; Bernerd, F.; Magnaldo, T. Genetic correction of stem cells in the treatment of inherited diseases and focus on xeroderma pigmentosum. *Int. J. Mol. Sci.* **2013**, *14*, 20019–20036.
9. Hainzl, S.; Peking, P.; Kocher, T.; Murauer, E.M.; Larcher, F.; Del Rio, M.; Duarte, B.; Steiner, M.; Klausegger, A.; Bauer, J.W.; et al. COL7A1 Editing via CRISPR/Cas9 in Recessive Dystrophic Epidermolysis Bullosa. *Mol. Ther.* **2017**, *25*, 2573–2584.
10. Suzuki, H.; Fukunishi, Y.; Kagawa, I.; Saito, R.; Oda, H.; Endo, T.; Kondo, S.; Bono, H.; Okazaki, Y.; Hayashizaki, Y. Protein-protein interaction panel using mouse full-length cDNAs. *Genome Res.* **2001**, *11*, 1758–1765.
